# Efficacy of a spatial repellent for control of *Aedes*-borne virus transmission: A cluster-randomized trial in Iquitos, Peru

**DOI:** 10.1073/pnas.2118283119

**Published:** 2022-06-23

**Authors:** Amy C. Morrison, Robert C. Reiner, William H. Elson, Helvio Astete, Carolina Guevara, Clara del Aguila, Isabel Bazan, Crystyan Siles, Patricia Barrera, Anna B. Kawiecki, Christopher M. Barker, Gissella M. Vasquez, Karin Escobedo-Vargas, Carmen Flores-Mendoza, Alfredo A. Huaman, Mariana Leguia, Maria E. Silva, Sarah A. Jenkins, Wesley R. Campbell, Eugenio J. Abente, Robert D. Hontz, Valerie A. Paz-Soldan, John P. Grieco, Neil F. Lobo, Thomas W. Scott, Nicole L. Achee

**Affiliations:** ^a^Department of Pathology, Microbiology, and Immunology, School of Veterinary Medicine, University of California, Davis, CA 95616;; ^b^Department of Virology and Emerging Infectious Diseases, US Naval Medical Research Unit No. 6, Lima, Peru, Washington, DC 20521-3230;; ^c^Department of Health Metrics Sciences, University of Washington, Seattle, WA 98195;; ^d^Department of Entomology and Nematology, University of California, Davis, CA 95616;; ^e^Dirección General de Saneamiento Ambiental, 16000 Iquitos, Peru;; ^f^Department of Entomology, US Naval Medical Research Unit No. 6, Lima, Peru, Washington, DC 20521-3230;; ^g^Genomics Laboratory, Pontificia Universidad Católica del Perú, San Miguel, Lima, Peru 15000;; ^h^Department of Global Community Health and Behavioral Sciences, Tulane School of Public Health and Tropical Medicine, New Orleans, LA 70112;; ^i^Department of Biological Sciences, Eck Institute for Global Health, University of Notre Dame, Notre Dame, IN 46556

**Keywords:** vector control, *Aedes aegypti*, spatial repellent, arbovirus vector, clinical trial

## Abstract

Over half the world’s population is at risk for viruses transmitted by *Aedes* mosquitoes, such as dengue and Zika. The primary vector, *Aedes aegypti*, thrives in urban environments. Despite decades of effort, cases and geographic range of *Aedes*-borne viruses (ABVs) continue to expand. Rigorously proven vector control interventions that measure protective efficacy against ABV diseases are limited to *Wolbachia* in a single trial in Indonesia and do not include any chemical intervention. Spatial repellents, a new option for efficient deployment, are designed to decrease human exposure to ABVs by releasing active ingredients into the air that disrupt mosquito–human contact. A parallel, cluster-randomized controlled trial was conducted in Iquitos, Peru, to quantify the impact of a transfluthrin-based spatial repellent on human ABV infection. From 2,907 households across 26 clusters (13 per arm), 1,578 participants were assessed for seroconversion (primary endpoint) by survival analysis. Incidence of acute disease was calculated among 16,683 participants (secondary endpoint). Adult mosquito collections were conducted to compare *Ae. aegypti* abundance, blood-fed rate, and parity status through mixed-effect difference-in-difference analyses. The spatial repellent significantly reduced ABV infection by 34.1% (one-sided 95% CI lower limit, 6.9%; one-sided *P* value = 0.0236, z = 1.98). *Aedes aegypti* abundance and blood-fed rates were significantly reduced by 28.6 (95% CI 24.1%, ∞); z = −9.11) and 12.4% (95% CI 4.2%, ∞); *z* = −2.43), respectively. Our trial provides conclusive statistical evidence from an appropriately powered, preplanned cluster-randomized controlled clinical trial of the impact of a chemical intervention, in this case a spatial repellent, to reduce the risk of ABV transmission compared to a placebo.

## Significance

Vector interventions are needed for *Aedes*-borne viral (ABV) disease prevention (dengue, Zika, chikungunya, and yellow fever), but their application is hindered by the lack of evidence proving they prevent infection or disease. We report conclusive statistical evidence from a pre-planned, prospective cluster-randomized, controlled clinical trial (cRCT) of protective efficacy (34.1% hazard estimate) against human ABV infection by a spatial repellent; a chemical-based intervention category currently under World Health Organization review. Results from our ABV study will help guide public health authorities responsible for operational management and worldwide ABV disease control and incentivize new strategies for disease prevention.

*Aedes*-borne viral diseases (ABVDs) [e.g., dengue (DENV), chikungunya, Zika (ZIKV), and yellow fever] are devastating, expanding global public health threats that disproportionally affect low- and middle-income countries. DENV, one of the most rapidly increasing vector-borne infectious diseases, results in ∼400 million infections each year (1, 2), with 4 billion people at risk for infection annually (3). Currently, the primary means for ABVD prevention is controlling the primary mosquito vector, *Aedes aegypti*. Existing vector control interventions, however, have failed to prevent ABV transmission and epidemics (4–6).

There is an urgent need to develop evidence-based guidance for the use of new and existing ABV vector control tools. The evidence base for vector control against ABVs is weak, despite considerable government investments in World Health Organization (WHO)-recommended control of larval habitats (larviciding, container removal) and ultra-low-volume insecticide spraying (4, 5, 7–9). These strategies continue to be implemented despite the lack of rigorously generated data from controlled clinical trials demonstrating they reduce ABV infection or disease (6). The only ABV intervention with a proven epidemiological impact in a cluster-randomized control trial (cRCT) assessed community mobilization to reduce mosquito larval habitats (10). A recent test-negative trial with *Wolbachia*-infected mosquitoes reported a significant reduction of DENV illness in Indonesia (11).

Spatial repellents (SRs) are devices that contain volatile active ingredients that disperse in air. The active ingredients can repel mosquitoes from entering a treated space, inhibit attraction to human host cues, or disrupt mosquito biting and blood-feeding behavior and, thus, interfere with mosquito–human contact (12–14). Any of these outcomes reduce the probability of pathogen transmission. Pyrethroid-based SRs have shown efficacy in reducing malaria infections in China (15) and Indonesia (16). There have, however, been no clinical trials evaluating the protective efficacy (PE) of SRs against ABV infection or disease.

To generate evidence for public health consideration, we conducted a double-blinded, parallel cRCT to demonstrate and quantify the PE of a transfluthrin-based SR to reduce ABV infection incidence over 2 y in a human cohort in Iquitos, Peru.

## Results

**Fig. 1.**
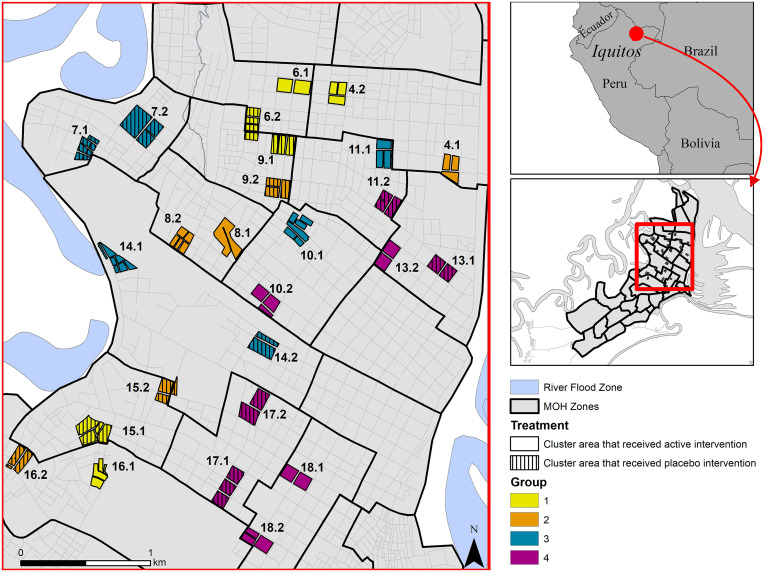
Location of 26 study clusters in Iquitos and Punchana Districts, Loreto Department, Iquitos, Peru. Each cluster consisted of approximately 140 households with an average distance of 523 m (range 280–879 m) between clusters.

**Fig. 2.**
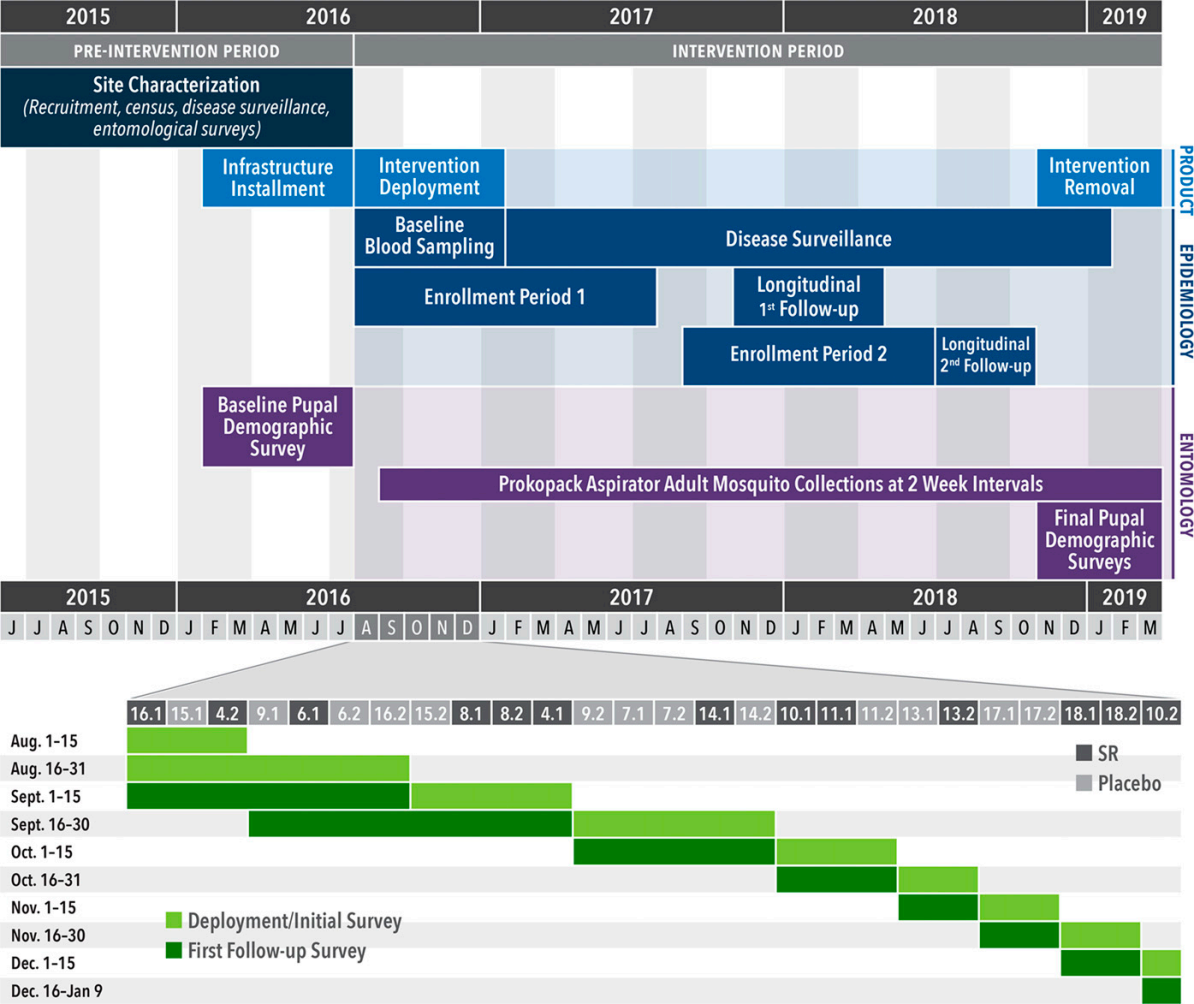
Study timeline. (*Top*) Human blood sampling, disease surveillance, and entomological monitoring in relation to deployment of the SR intervention. (*Bottom*) Intervention rollout between Aug. and Dec. 2016 by cluster. Horizontal numbers correspond to cluster numbers shown in Fig. 1.

We report results from the intervention phase of a cRCT, conducted in 26 clusters (13 per arm; see *Methods* and *SI Appendix*, section 1.2.1 for randomization scheme) and each with ∼140 households (60 qualifying participants), between Aug. 2016 and Mar. 2019 (Figs. 1 and 2 and *SI Appendix*, section 1.1).

The primary endpoint was ABV seroconversion, as measured by DENV- or ZIKV-specific neutralizing antibodies, in blood from children ≥2 to ≤18 y, collected just prior to the deployment of the SR intervention, and ∼1 and 2 y later. The SR intervention was a transfluthrin passive emanator placed in participating households according to manufacturer’s instructions: one product per 9 m^2^ and replaced at 15-d intervals (*SI Appendix*, Fig. S1 and section 1.3.2) during the 2-y (two-transmission seasons) study period. Secondary endpoints were clinically apparent laboratory-confirmed ABVD and indoor female *Ae. aegypti* abundance, blood-fed status (proxy for human-biting rates), and parity status (proxy for age structure). Participants followed for seroconversion were the “longitudinal cohort,” and those followed for disease were the “febrile surveillance cohort.”

### Study Population.

**Fig. 3.**
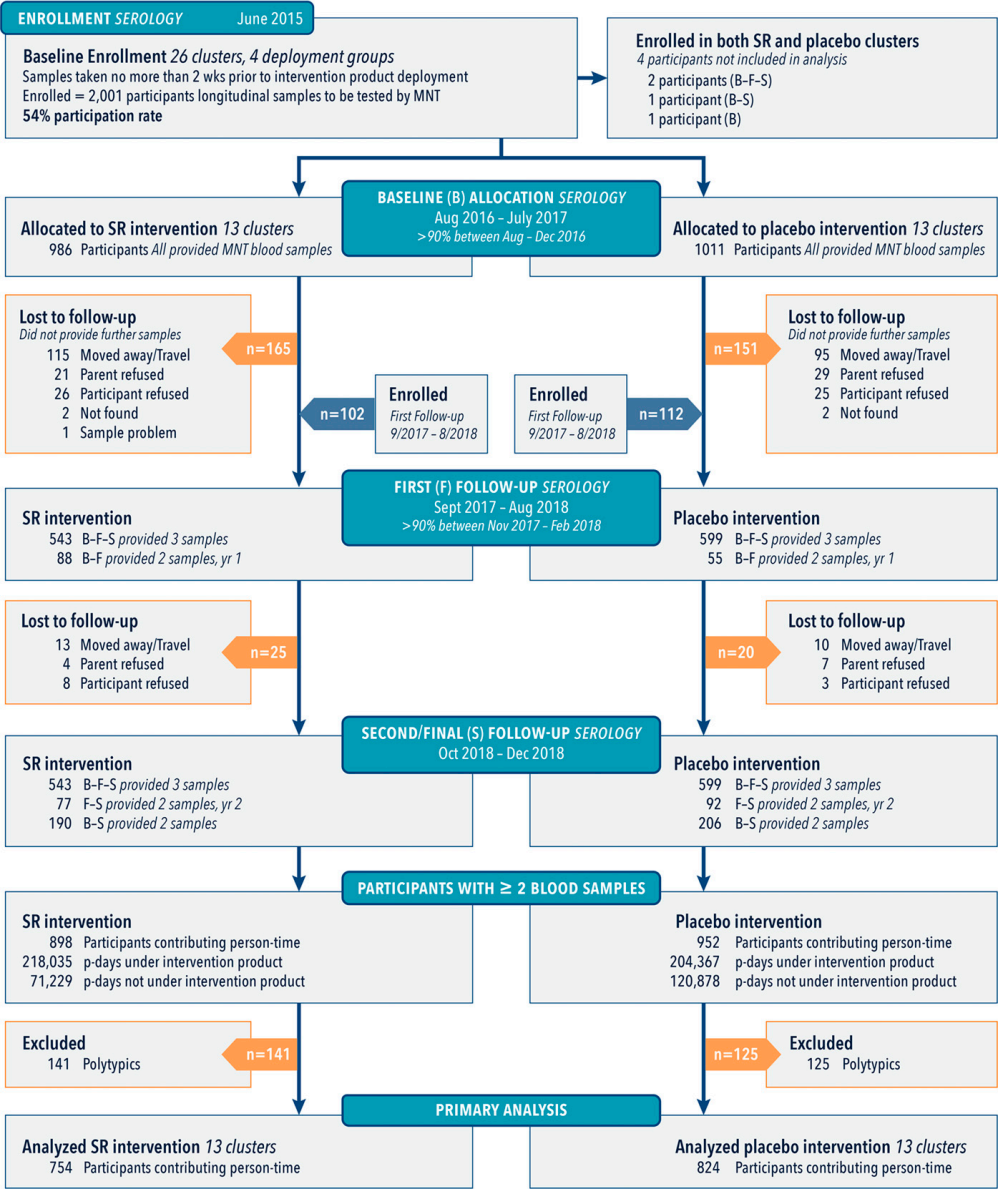
Allocation and follow-up of the longitudinal cohort population during three blood collection periods (baseline [B], first [F], and second/final [S]). The majority (62%) of participants provided samples at each collection period, whereas some only participated during year 1 (B-F) or year 2 (F-S). Participants with a single sample were lost to follow-up, and four individuals moved or had two houses located in the SR. Placebo clusters are shown as removed at the baseline period for clarity.

A total of 2,215 persons were enrolled in the longitudinal cohort. Of these, 1,578 qualifying participants (individuals who were seronegative or had a monotypic DENV antibody response when they entered the trial) were included in the intent-to-treat (ITT) analysis for seroconversion (Fig. 3).

Samples were tested by microneutralization enzyme immunoassay (MNT) for seroconversion to each DENV serotype and ZIKV (*SI Appendix*, sections 1.3.3.1 and 1.3.4.1). Only participants who provided at least two blood samples were included in final analyses. We observed a total of 196 ABV infections from 754 (1,090 paired samples) qualifying participants in the SR arm and 294 ABV infections from 824 (1,237 paired samples) qualifying participants in the placebo arm. Baseline covariates were balanced at both the cluster and individual levels (Table 1).

**Table 1.** Summary of baseline characteristics, for qualifying participants of the ITT longitudinal cohort population in SR and placebo arms.

Individual level		
Variable	SR	Placebo
	(*n* = 898)	(*n* = 952)
Age in years (mean ± SD)	10.88 ± 4.62	10.19 ± 4.39
(min, max)	(2, 47)	(2, 22)
Sex (% male)	51.6%	55.0%
Duration in years between samples (mean ± SD)	1.15 ± 0.42	1.16 ± 0.43
(min, max)	(0.02, 2.29)	(0.01, 2.35)
No. qualifying participant observations	1,090	1,237
No. arbovirus seroconversions	196	294

Cluster level		
Variable	SR	Placebo
	(*n* = 13)	(*n* = 13)
Cluster population (mean ± SD)	58.2 ± 30.4	73.5 ± 35.3
(min, max)	(21, 124)	(12, 132)
Baseline susceptibility (mean ± SD)	81.0 ± 12.8%	84.9 ± 6.3%
(min, max)	(54.7, 98.1%)	(73.3, 92.5%)

Qualifying participants were defined as individuals in a participating house that were seronegative or had a monotypic DENV antibody response when they entered the trial.

A total of 16,707 participants were followed for clinical disease in the febrile surveillance cohort through ∼3 wellness visits per week, of which 16,683 were included in the ITT analysis (*SI Appendix*, Fig. S2). Suspected acute ABV cases that provided consent provided acute and convalescent blood samples and were monitored clinically daily. Acute serum samples were tested for viral RNA by PCR (DENV and ZIKV; *SI Appendix*, sections 1.3.3.2–1.3.3.4) and by enzyme-linked immunosorbant assay (ELISA) for DENV immunoglobulin M (IgM) (*SI Appendix*, sections 1.3.3.5 and 1.3.4.2) to quantify the secondary endpoint of clinically apparent laboratory confirmation cases of ABVD. Baseline covariates from the febrile surveillance cohort were balanced at both the cluster and individual levels (*SI Appendix*, Table S4).

### Intervention Coverage.

The household participation rate (intervention deployed at some point during the study period) per cluster (SR and placebo) was 56.6% (SD = 10.5%), with slightly more participation in SR than in placebo clusters (58.8 vs 54.5%, *P* value = 0.336). In households consenting to receive intervention (SR or placebo), the mean percentage of days covered by an intervention at the cluster level was 81.6% (SD = 3.9), with slightly higher coverage in households assigned to SR intervention (82.9%) compared to households in the placebo arm (80.3%), albeit insignificant (*P* value = 0.153; *SI Appendix*, Table S2 and Fig. S3). For all enrolled households, the mean percentage of days with an adequate intervention application rate (one product per 9 m^2^) was 73.6% (SD = 9.1), with similar rates between SR and placebo clusters (72.7 vs 74.5%, *P* value > 0.999; *SI Appendix*, Table S2).

### SR Efficacy.

We used survival analysis with proportional hazards model with an exponential distribution assumption for baseline hazard to estimate a PE (*SI Appendix*, section 1.5.2.1). The estimated PE of the SR intervention was 34.1% (one-sided 95% CI lower limit, 6.9%) (Table 2). Reduction in the arbovirus infection hazard rate was significant at the 5% significance level (test statistic: *z* = 1.98, one-sided *P* value = 0.02). Baseline covariates included in the statistical model on the hazard of arbovirus infection in qualifying participants were age—which had statistically significant effects on the hazard of arbovirus infection in qualifying participants—and sex, which did not. Reported age- and sex-specific hazard rate changes are conditional. Hazard rate increases by 4.6% for every 1-y increase in age. Hazard rate decreases by 4.4% in males relative to females (Table 2). The originally proposed ITT mixed-effects logistic regression analysis (*SI Appendix*, section 1.5.2.2), which ignores differential participation duration across qualifying participants, produced a result consistent with those presented here (i.e., PE >30% and statistical significance at the 5% level; Table 2).

**Table 2.** PE estimates from ITT analyses for the SR intervention against ABV infection in qualifying participants measured by seroconversion (primary endpoint), including covariate effects.

ITT analysis	HR ratio	PE (%)	One-sided	Covariate	Odds ratio	Two-sided
	(95% CI)	(95% CI)	*P* value		(95% CI)	*P* value
Survival analysis	0.659	34.1	0.024			
	(−∞, 0.931)	(6.9, ∞)				
				Age	1.046	6.8 x 10^−6^
					(1.029 to 1.063)	
				Male	0.956	0.62
					(0.821 to 1.112)	

ITT analysis	Odds ratio	PE (%)	One-sided	Covariate	Odds ratio	Two-sided
	(95% CI)	(95% CI)	*P* value		(95% CI)	*P* value
Odds reduction	0.547	45.3	0.019			
	(−∞, 0.883)	(11.7, ∞)				
				Age	1.071	6.9 x 10^−7^
					(1.042 to 1.100)	
				Male	0.950	0.64
					(0.767 to 1.177)	
Odds reduction on including 9- to 15-mo sampling intervals	0.577	42.3	0.016			
	(−∞, 0.879)	(12.1, ∞)				
				Age	1.065	0.0005
					(1.028 to 1.103)	
				Male	0.970	0.779
					(0.767 to 1.299)	

**Fig. 4.**
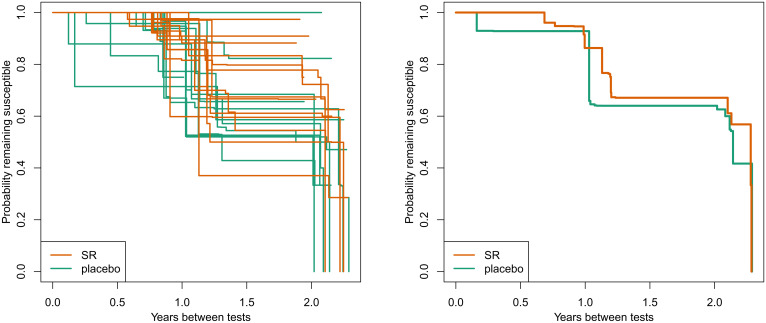
Kaplan-Meier curves of arbovirus infection for 13 SR and 13 placebo clusters in qualifying participants measured by seroconversion (primary endpoint) by cluster. (*A*) Hazard rates by individual cluster. (*B*) Aggregated hazard rate.

The Kaplan-Meier curves of arbovirus infection for qualifying participants by cluster show considerable between-cluster variation (SR and placebo clusters), as evidenced by the wide spread of survival curves (Fig. 4).

For example, there were no arbovirus infections in qualifying participants in placebo cluster 7.2, which had only 18 qualifying participants. Conversely, of the 11 qualifying participants who had a duration of at least 15 mo between tests in cluster 8.2, a total of 5 subjects became infected since their last test. The duration between tests varied by participant and across clusters (*SI Appendix*, Fig. S4), resulting in some Kaplan-Meier curves being estimated beyond 2 y. In many of those clusters, the only participants that went over 2 y between blood sampling were universally found to have had an arboviral infection.

A Poisson generalized linear regression was used to assess intervention impact on clinical disease, with an offset for the number of participant days each participant spent in each cluster. No covariates were used, and due to the small sample size, no random effects were incorporated (*SI Appendix*, section 1.5.3). No statistical difference between incidence in the SR and placebo arms was detected. Baseline characteristics of covariates included in the analysis of PCR/ELISA confirmed DENV and ZIKV cases were balanced between SR and placebo arms (*SI Appendix*, Table S5). Results from an ITT fixed-effect Poisson generalized linear model indicate the rate ratio is 1.144, with an upper bound on the one-sided 95% CI of 1.601. This translates into a 14.4% increase in the rate of PCR/ELISA-confirmed arbovirus infections by SR intervention compared with placebo, with the lower bound of the one-sided 95% CI of –60.1%. This apparent increase in the intervention area was not statistically significant at the 5% level (test statistic: z= −0.975), in part because only 96 disease cases were detected—51 in the SR arm and 45 in the placebo arm—during 10,793,792 participant days that appeared balanced between SR and placebo clusters (*SI Appendix*, Table S4 and section 2.5.2.1).

**Fig. 5.**
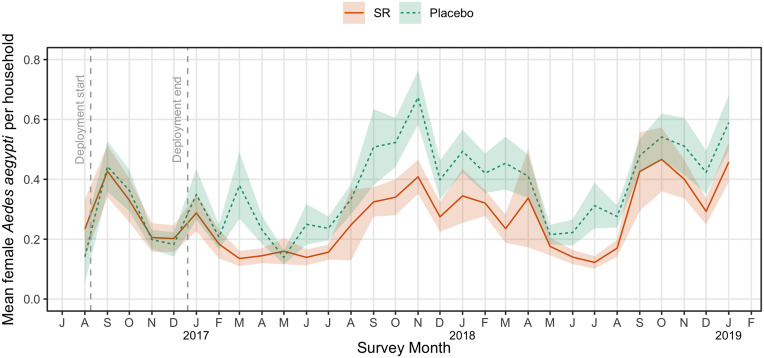
Mean densities of adult female *Aedes aegypti* collected per household survey in 13 SR and 13 placebo clusters by study month. Shaded areas represent the 95% CI around the mean.

The estimated reduction in adult female *Ae. aegypti* abundance in clusters receiving SR intervention was 28.6% (one-sided 95% CI lower limit: 24.1%, test statistic: *z* = −9.11) using mixed-effects difference-in-difference (DID) Poisson regression with factor-level covariates (Table 3 and *SI Appendix*, section 1.5.4). Baseline mosquito abundance was balanced between treatment arms (*SI Appendix*, Table S5) with postbaseline quantities estimated based on 47,518 and 43,417 household collections in SR and placebo arms, respectively. Baseline abundance averaged 0.277 (SD 0.153) and 0.279 (SD 0.122) per house survey in SR and placebo arms, respectively, whereas postbaseline abundance averaged 0.276 (SD 0.091) and 0.391 (SD 0.142) in the SR and placebo arms, respectively (Table 3). There was strong indication of seasonality, with estimated *z*-scores of 6 or greater when comparing each month to the reference month of January (*SI Appendix*, Table S7). Overall, abundance trended lower in the SR clusters compared to the placebo clusters, after intervention deployment for the duration of the trial from 2017 to 2019 (Fig. 5).

**Table 3.** Rate reduction summary of indoor adult female *Aedes aegypti* abundance, blood-fed female abundance, and parity rates (secondary endpoints) for primary (ITT) and secondary analyses (PP).

Indicator	Statistics	2016 baseline*		Cluster-specific baseline^†^	
		ITT	PP	ITT	PP
Indoor adult female *Ae. aegypti* abundance	Rate ratio	0.714	0.737	0·599	0.607
	(95% one-sided CI)	(−∞, 0.759)	(−∞, 0.784)	(−∞, 0.633)	(−∞, 0.641)
	Rate reduction (%)	28.6	26.3	40.1	39.3
	(95% one-sided CI)	(24.1, ∞)	(16.2, ∞)	(36.7, ∞)	(35.9, ∞)
Blood-fed female *Ae. aegypti* abundance	Rate ratio	0.876	0.876	0.908	0.929
	(95% one-sided CI)	(−∞, 0.958)	(−∞, 0.958)	(−∞, 0.985)	(−∞, 1.06)
	Rate reduction (%)	12.4	12.4	9.20	7.10
	(95% one-sided CI)	(4.2, ∞)	(4.2, ∞)	(1.49, ∞)	(−6, ∞)
*Ae. aegypti* parity rate	Rate ratio	0.922	0.921	0.928	0.909
	(95% one-sided CI)	(−∞, 1.057)	(−∞, 1.056)	(−∞, 1.058)	(−∞, 0.986)
	Rate reduction (%)	7.75	7.87	7.24	9.10
	(95% one-sided CI)	(−5.70, ∞)	(−5.60, ∞)	(−5.80, ∞)	(−1.2, ∞)

No correction for multiple testing was performed for secondary endpoint analyses, and, as such, in accordance with CONSORT guidelines, *P* values are not presented.

^*^ITT (primary analysis) and per protocol (PP, secondary analysis) mixed-effects DID Poisson regression specifying measurements made throughout 2016 as “baseline,” with post-2016 measurements as “postbaseline.” PP analysis only considering houses with SR application rates meeting manufactures specifications ≥75% of the days between sequential blood sampling (designated PP-1).

^†^ITT and PP mixed-effects DID Poisson regression specifying measurements made in 2016 up to the first date of intervention deployed in each cluster as “baseline,” with all measurements following that date as “postbaseline” for that cluster, even for houses that did not enroll until 2017 or later. PP analysis only considering houses with SR application rates meeting manufacturer’s specifications ≥75% of the days between sequential blood sampling (designated PP-1).

Postintervention entomological surveys indicate that this difference disappeared after removal of the intervention (*SI Appendix*, Fig. S8*B*).

The estimated reduction in the rate of blood-fed *Ae. aegypti* collected inside houses was 12.4% (one-sided 95% CI lower limit: 4.2%, test statistic: *z* = −2.430), also using mixed-effects DID Poisson regression (Table 3). Baseline abundance of engorged *Ae. aegypti* was balanced across treatment arms (*SI Appendix*, Table S5), with postbaseline quantities estimated based on 9,257 and 11,496 mosquitoes assessed for blood-fed status in SR and placebo arms, respectively. Baseline abundance of blood-fed mosquitoes averaged 0.593 (SD 0.440) and 0.536 (SD 0.451) per collection in SR and placebo arms, respectively. Postbaseline rates averaged 0.606 (SD 0.460) and 0.634 (SD 0.447) in SR and placebo arms, respectively. There was no strong indication of seasonality (*SI Appendix*, Table S8).

There was no observed intervention effect (Table 3) or indication of seasonality (*SI Appendix*, Table S9) based on the parity rate of *Ae. aegypti* females.

### AEs and SAEs.

In total, 29 adverse events (AEs) were reported during the trial (*SI Appendix*, section 2.8). Of these, three were associated with blood draw (vasovagal response: two from SR and one from placebo clusters). The remaining 26 AEs reflected symptoms consistent with pyrethroid/transfluthrin exposure. Reporting of these AEs was higher in the SR clusters (22 of 8,235 subjects: 0.267%) than the placebo clusters (4 of 8,448 subjects: 0.047%). The relative risk of experiencing a mild AE due to the SR product was 5.6 (95% CI: 1.9 to 16, *P* = 0.0015). The 26 affected individuals (26 of 16,707 subjects; 0.155%) came from 18 households, often reporting different combinations of the following symptoms: allergic response with itching and skin irritations (*n* = 19; 15 from SR, 4 from placebo clusters); dry mouth/bad taste (*n* = 5, from SR clusters only); breathing issues (*n* = 7, from SR clusters only), including exacerbation of chronic bronchitis or asthma (*n* = 2); and headaches (*n* = 4, from SR clusters only). The total number of AEs reported from longitudinal subject cohort households during the trial was similarly low (18 of 2,907 households; 0.619%). No reported serious AEs (SAEs) (deaths) were deemed associated with the SR intervention.

## Discussion

ABVs have expanding regions of transmission, cause increasingly frequent epidemics, and are transmitted by one of the most anthropophilic mosquitoes. There is, therefore, a growing unmet need for effective ABVD prevention (9). Our cRCT provides conclusive statistical evidence from a preplanned, prospective cRCT of significant PE (34.1% hazard estimate) against human ABV infection by a chemical-based vector control intervention, in this case, a SR. The uniqueness of our trial in the realm of *Aedes* vector control is that it is a preplanned cRCT with 1) a predefined effect size on the primary endpoint of human infection that 2) was appropriately powered to prospectively quantify and statistically test for a difference in the impact of a chemical intervention against ABV human infection incidence compared to a placebo. Current adult *Aedes* chemical control strategies are not supported by such evidence. Reduced human infection caused by a reduction in mosquito biting supports development of 1) improved repellent formulations and 2) enhanced methodologies for broad-scale application.

PE was detected despite assumed dilution and contamination effects due to participant movement in and out of study clusters. Unlike a vaccine, SR protected study participants in their own homes or another protected home within their neighborhood (i.e., study cluster) but did not provide continuous protection after they left treated houses. Our cohort, tested for ABV seroconversion, comprised principally children ≤17 y of age, most of whom attended schools that have been shown in Iquitos to be of lower risk of *Ae. aegypti* infestation than residential sites (17). Our outcome was demonstrated in an operational context, reflecting complex interactions among ongoing Ministry of Health interventions across the study area, imperfect coverage at the household level (rooms closed to intervention, homeowner removal, and/or loss of intervention), <100% household participation within clusters, and the suggestion of pyrethroid resistance in the local *Ae. aegypti* population (18) (*SI Appendix*, section 2.7). Reduced risk of ABV infection was associated with a significant reduction in indoor female *Ae. aegypti* abundance and blood feeding. Although entomological outcomes were modest, detected effects are consistent with the expected mode of SR action (i.e., deterrence from house entry and/or interfering with human biting) (12, 19), and the impact waned after the intervention was removed at the end of the trial.

Our results support SRs as a flexible class of vector control products with positive public health impact not limited to ABV diseases. Transfluthrin- (15) and metofluthrin-based (20) mosquito coils have been shown to reduce malaria, and the same SR device used in our Iquitos trial reduced malaria infections in an Indonesia cRCT (16). The SR product we tested was generally well tolerated even though it produced mild skin and respiratory irritation, a well-known side effect of pyrethroids. Our trial quantifies these types of AEs for a chemical intervention in a double-blinded trial. Our results, therefore, support the potential for SRs to reduce a variety of vector-borne diseases, augment existing public health efforts, and support SRs as an effective component in vector control intervention strategies. To facilitate implementation and programmatic scale-up, additional assessments, which have already begun (21–24), are needed.

Our Peru cRCT is one of two trials recommended by the WHO for assessing public health value and developing global health policy for the SR intervention class (25). Our study was powered to detect a 30% reduction in ABV infection risk, not acute ABVD or virus infection rates in mosquitos. During the trial period, DENV prevalence was lower than in previous years, and a ZIKV epidemic occurred in 2016 (26). This epidemiological uncertainty is typical of ABV transmission, making powering ABV cRCTs challenging, and helps explain why cRCTs with epidemiological outcomes for ABVs are rare (27). We used seroconversion as our primary endpoint of PE to address this challenge. At our Iquitos study site, powering a trial based on clinically apparent ABVD would not be logistically feasible.

Fully integrating vector control into ABVD prevention programs will require quantitative guidance based on quantitative measures of the impact from each intervention component. Ministries of Health, local to national governments and nongovernmental organizations, can use our trial results as an evidence base for informed application of SRs. Considering the growing ABV public health threat, difficulties of developing vaccines against multiple viruses, and past poorly informed vector control failures (6), enhanced ABVD prevention will benefit greatly from interventions with proven public health value.

## Materials and Methods

This trial is registered with ClinicalTrials.gov, number NCT03553277.

### Ethical Statement.

Our study protocol (#NAMRU6.2014.0021; *SI Appendix*) was approved by the US Naval Medical Research Unit No. 6 (NAMRU-6) Institutional Review Board (IRB), which includes Peruvian representation and complies with US Federal and Peruvian regulations governing the protections of human subjects, and the Regional Health Authority (DIRESA), the local branch of the Peruvian Ministry of Health. IRB authorization agreements were established among the NAMRU-6; the University of Notre Dame (sponsor); the University of California, Davis; and the University of Washington.

### Trial Design.

Detailed study methods are provided in *SI Appendix*.

Our trial was conducted from Jun. 2015 through Mar. 2019 in the Iquitos and Punchana Districts of Iquitos, Peru (Fig. 1 and *SI Appendix*, section 1.1). Clusters were selected in Jan. 2015. Enrollment began Jun. 2015. Participation included 1) a house census, 2) disease surveillance, 3) annual blood draws, 4) bimonthly entomological surveys, and 5) intervention application in the house. Epidemiological monitoring and entomological surveillance lasted from Feb. 2016 through Mar. 2019 (Fig. 2).

Our main objective was to demonstrate and quantify the PE of an SR in reducing ABV infection incidence in a human cohort. Qualifying participants were individuals in a participating house who were seronegative or had a monotypic DENV antibody response when they entered the trial. Assuming the probability of seroconversion for seronegative or monotypic individuals was 10% with a coefficient of variation of 0.25 and an alpha of 5%, we estimated we would need 26 clusters (13 per arm) with ∼60 qualifying individuals to achieve a power of 80% to detect a reduction in the odds of 30%.

The primary endpoint was ABV seroconversion, as measured by DENV- or ZIKV-specific neutralizing antibodies, in blood from children ≥2 to ≤18 y. To increase the pool of baseline seronegative participants, we expanded screening to ≥18 y. Secondary endpoints were clinically apparent, laboratory-confirmed ABVD and indoor female *Ae. aegypti* 1) abundance, 2) blood-fed status (proxy for human-biting rates), and 3) parity status (proxy for age structure). Participants followed for seroconversion were the “longitudinal cohort,” and those followed for disease were the “febrile surveillance cohort.”

### Randomization and Intervention.

A total of 26 clusters (13 per arm), each with ∼140 households (60 qualifying participants), were randomly allocated in Aug. 2016 to receive SR or placebo intervention by the external statistician serving on the Data Safety Monitoring Board (DSMB) using a random number generator (https://www.random.org) (*SI Appendix*, section 1.2.1). Investigators, research staff, and study participants were blinded to cluster allocation. Our intervention was a transfluthrin passive emanator designed and produced by SC Johnson (Racine, WI), replaced at 2-wk intervals, as described previously (*SI Appendix*, Fig. S1 and section 1.3.2) (16). Our trial was specifically designed to evaluate the PE of the first in-class SR product prototype to enable WHO assessment for public health value of the SR product class. SR products are not yet recommended by WHO for inclusion into programmatic vector control strategies. We selected the SC Johnson–manufactured, transfluthrin-based passive emanator because it represents the first in-class prototype of a SR under WHO public health value assessment (28). The SR intervention used in our study is the same intervention evaluated against malaria infections in Sumba, Indonesia (16). SR and placebo intervention had identical packaging and were deployed in houses by study personnel using a blinded coding scheme. The placement of the intervention followed manufacturer specifications for indoor use conditions.

### Seroconversion and Disease Surveillance.

Recruitment for the longitudinal cohort focused on children because they were more likely to be antibody test negative or monotypic at baseline than adults, which would facilitate interpretation of laboratory assays, and less mobile than adults (29), thus spending more time in their houses or their assigned cluster. Baseline blood samples were obtained within 2 wk before or after initial intervention deployment. As new families moved into the study area, they were recruited to participate, resulting in longitudinal participant enrollment throughout the interval among baseline (B), first (F), and second/final (S) longitudinal blood draws (Fig. 3). Samples were tested by MNT for seroconversion to each DENV serotype and ZIKV (*SI Appendix*, sections 1.3.3.1 and 1.3.4.1). Only participants who provided at least two blood samples were included in final analyses.

The febrile surveillance cohort was recruited by nurse technicians during door-to-door wellness checks starting with the first week of intervention deployment. Suspected cases exhibited axillary temperature of ≥37.5 °C or, for suspected ZIKV infection, absence of fever but presence of rash, arthralgia, arthritis, or nonpurulent conjunctivitis for ≤5 days. Participants meeting these criteria provided acute and convalescent (14 to 21 d later) serum samples and were monitored clinically daily. Acute serum samples were tested for viral RNA by PCR (DENV and ZIKV; *SI Appendix*, sections 1.3.3.2–1.3.3.4) and by ELISA for DENV IgM (*SI Appendix*, sections 1.3.3.5 and 1.3.4.2).

### Entomological Endpoints.

Indoor Prokopack aspirations (30) were conducted in all consented homes at time of first intervention deployment and subsequent intervention replacement (i.e., 2-wk intervals). Adult mosquitoes were transported to the NAMRU-6 Iquitos laboratory, sedated at 4 °C, identified to species and sex, and counted by date and house. Up to 30 female *Ae. aegypti* per household per collection were examined for bloodmeal status and scored as unfed, blood-fed (fully engorged, half-engorged, or trace amounts), or gravid (31). These female mosquitoes were then dissected to determine their parity status (parous, nulliparous, or gravid) (*SI Appendix*, section 1.3.5.1). Standard insecticide resistance assays were used to assess vector susceptibility to transfluthrin 1 y into the trial (*SI Appendix*, section 1.3.5.2).

### Safety Monitoring.

AEs and SAEs were actively collected throughout the trial during surveillance follow-up and entomological surveys (*SI Appendix*, section 1.3.6). Reported AEs were investigated by study staff, and appropriate care was recommended by a study physician within 24 h. Safety reporting to the NAMRU-6 IRB was managed by UC Davis in accordance with the approved protocol. Quarterly reports summarizing reported AEs and SAEs were reviewed by the DSMB for trial safety assessment.

### Statistical Analysis.

Details of our analytical approach are provided in the statistical analysis plan (SAP) and *SI Appendix*, section 1.5. All analyses were conducted by Robert C. Reiner Jr. using R 3.6.1 (R Core Team) (32) and the Ime4 (33) and survival (34) packages. No correction for multiple testing was performed for secondary endpoint analyses, and, as such, in accordance with the Consolidated Standards of Reporting Trials (CONSORT) guidelines, in those cases we do not present *P* values.

The choice of the investigators to select and report a one-sided *P* value and corresponding one-sided CI is based on the underlying assumption associated with the intervention (i.e., the SR will not increase risk/harm [disease] over the standard of care). The statistical translation is that we are testing to see if 1) the intervention is no better than the standard of care or 2) the intervention is better than the standard of care (superior). As this is a one-sided test of superiority, we present one-sided test results. We also provide the null and alternative hypotheses, test statistic, and its assumed distribution to provide readers information to interpret the *P* value and confidence interval.

The primary analysis was an ITT assessment of ABV seroconversion for all qualifying participants per treatment assignment who were >2 to <18 y of age. Due to the rolling nature of enrollment, we used a survival analysis with a proportional hazards model and exponential distribution assumption for the baseline hazard (i.e., constant baseline hazard through time) and a frailty component to account for correlation within clusters. If $h(t_{ij}|x_{ij})$ is the hazard rate of the $j^{th}$ individual in the $i^{th}$ cluster with covariate values $x_{ij}$, then this individual’s hazard rate of an arbovirus infection can be written as

$$h(t_{ij}|x_{ij})\;=h_{0}\;(t_{ij})\;\cdot \exp\;(\beta^{T}x_{ij}+W_{i})$$

where $W_{i}∼N(0,\sigma_{c}^{2})$ is the random effect of the $i^{th}$ cluster. Covariates included age, sex, and treatment status (SR or placebo). PE was estimated as $PE=\left(1-\exp\;\left(\hat{\beta }\right)\right)\times 100\%$, where $\hat{\beta }$ is the estimated regression coefficient for the intervention group, and $\exp\;\left(\hat{\beta }\right)$ is the estimated hazard ratio (HR) between SR and placebo. The null hypothesis of PE = 0% is equivalent to β=0, which is tested by Wald’s test z=β/s, where s is the estimated SE of $\hat{\beta }$, at the one-sided significance level of 5%.

A Poisson generalized linear regression was used to assess intervention impact on clinical disease, with an offset for the number of participant days each participant spent in each cluster. No covariates were used, and due to the small sample size, no random effects were incorporated.

Indoor adult female *Ae. aegypti* abundance, blood-fed rates, and parity were tested through DID Poisson generalized linear mixed models. Collections conducted in 2016 were defined as “baseline,” and collections conducted in 2017 and 2018 were estimated as “postbaseline.” Each analysis accounted for month of year and year as fixed effects and contained a random effect by cluster. Model formulation details are presented in *SI Appendix*, sections 1.5.4–1.5.6.

The primary analysis conducted differs from that in the original SAP (SI SAP). Procedures called for yearly blood draws from children. To increase the number of qualifying participants, we expanded the age range of the longitudinal cohort to all ages ≥2 y. The rolling enrollment resulted in substantial variation in time intervals between blood draws. Simple logistic regression, therefore, was inadequate. The decision to alter the primary analysis was discussed and agreed upon by the study statistician and the external DSMB statistician before outputs were unblinded to the DSMB statistician.

## Supplementary Material

Supplementary File

## Data Availability

All analytical data sets have been deposited in CurateND (https://doi.org/10.7274/xk81jh37s8g). Data include anonymized sr_febrile_cases.csv, sr_location_ent.csv, sr_longitudinal.csv, sr_questionanaire.csv, and the folders: SAE data as submitted in final DSMB report and preintervention_entomology. Analytical codes are provided in the *SI Appendix* (SAP) as well as in the data repository.
